# The Hha-TomB Toxin-Antitoxin System Shows Conditional Toxicity and Promotes Persister Cell Formation by Inhibiting Apoptosis-Like Death in *S.* Typhimurium

**DOI:** 10.1038/srep38204

**Published:** 2016-12-02

**Authors:** Sangeeta Jaiswal, Prajita Paul, Chandrashekhar Padhi, Shilpa Ray, Daniel Ryan, Shantoshini Dash, Mrutyunjay Suar

**Affiliations:** 1 School of Biotechnology, KIIT University, Bhubaneswar-751024, Odisha, India

## Abstract

Toxin-antitoxin (TA) modules are two component “addictive” genetic elements found on either plasmid or bacterial chromosome, sometimes on both. TA systems perform a wide range of functions like biofilm formation, persistence, programmed cell death, phage abortive infection etc. *Salmonella* has been reported to contain several such TA systems. However, the hemolysin expression modulating protein (Hha) and its adjacent uncharacterized hypothetical protein TomB (previously known as YbaJ), have not been listed as a TA module in *Salmonella*. In this study we established that Hha and TomB form a bonafide TA system where Hha serves as a toxin while TomB functions as an antitoxin. Interestingly, the toxicity of Hha was conditional causing cell death under acid stress. The antitoxin attenuated the toxicity of Hha by forming a TA complex through stable interactions. The Hha-TomB TA system was found to increase persistence and inhibit programmed cell death under antibiotic stress where a phenotypically diverse population expressing differential level of TA components was observed. Therefore we propose that Hha and TomB prevent cells from committing suicide thereby promoting persister cell formation.

Genomes of prokaryotes contain small genetic modules encoding a stable toxin and a counteracting, labile antitoxin^1^,^2^. Such a module, known as a toxin antitoxin (TA) system has been linked to several processes like persistence^3^, multidrug tolerance in bacteria^4^, biofilms^5^, stress response^6^, virulence^5^,^7^,^8^ and programmed cell death^9^. The toxin of a TA system that has been shown to target several cellular processes such as DNA replication, protein synthesis, RNA degradation etc., is most likely a mediator for such functions^1^. Depending on the molecular nature (RNA or DNA) and role of the antitoxin, TA systems are of five classes. Antitoxins of Type I and type III TA systems are RNA molecules that counteract the action of the toxin either by regulating toxin gene expression (Type I) or by forming a stable toxin antitoxin complex (Type III)^2^,^10^. Type II, IV and V antitoxins are proteins of which type IV functions as an antagonist and competes with the toxin for target binding^11^, while type V are endoribonucleases which cleave the transcript of the toxin^12^ and type II antitoxins bind directly to their cognate toxins and neutralize their activity. According to previous reports, type II toxin and antitoxin proteins are operonic and the antitoxin has been shown to repress transcription of the operon by binding to a certain palindromic stretch present within the promoter^13^. Certain stimuli induce degradation of the antitoxin by proteases; releasing free toxin which relieves transcriptional repression of the operon maintaining high levels of free toxin until the stimuli for the degradation of the antitoxin is withdrawn. A comprehensive genome analysis of various bacteria revealed the presence of several unnoticed type II TA modules^13^. TA systems are more abundant in pathogenic bacteria as compared to their non-pathogenic counterparts suggesting their role in bacterial virulence^14^. Hence, identification of new TA modules in pathogenic bacteria will enhance our knowledge on bacterial virulence.

*Salmonella* are Gram negative enteropathogenic bacteria which cause enteric diseases in humans and other hosts. Recent studies have established the presence of several type I and type II TA systems in *Salmonella enterica* serovar Typhimurium (*S.* Typhimurium)^8^,^15^. Further, De La Cruz *et al*. (2013) reported that the non-pathogenic species of *Salmonella* i.e. *S.* bongori does not contain any type II TA system while pathogenic *S.* Typhimurium contains more than 15 type II TA system^8^ one of which (SehAB) plays an important role in survival within lymphoid organs during infection in mice^8^. Moreover, one study has reported that the ShpAB TA system of *S.* Typhimurium plays an important role in persistence^8^. Using several bioinformatics tools, two independent studies identified 11 and 19 type II TA modules in *S.* Typhimurium^8^,^15^. However, neither of the studies identified the hemolysin expression modulating protein, Hha and its adjacent protein TomB as a TA module. Hha is a small (8.79 kDa) nucleoid associated protein belonging to Hha-YmoA family of proteins which are actively involved in gene regulation in Gram-negative bacteria^16^. It interacts directly with the H-NS protein and regulates expression of horizontally acquired genes in Enterobacteria^17^,^18^,^19^. Furthermore, Hha has been shown to be required for persister cell formation in *E. coli*^20^. In *S.* Typhimurium, Hha negatively modulates the expression of *hilA*, a transcriptional activator of SPI-1 genes^21^ while no functional role has been assigned to TomB till date. In the present study we have established that, Hha and TomB bear the characteristics of a bonafide type II TA system and investigated its role in persistence and programmed cell death (PCD) in *S.* Typhimurium.

## Results

### Transcriptional regulation of *hha* and *tomB* promoter

The arrangement of *hha* and *tomB* on the *S.* Typhimurium genome was determined from the National Center for Biotechnology Information (NCBI) database wherein both the genes were found to be on the negative strand, just 28 bp apart. The *hha* gene encodes an 8.79 kDa protein while *tomB* encodes a 13.64 kDa protein (Fig. S1a). BPROM predicted 3 putative promoters namely p201, p622 and p922 (Fig. S1b). The program also predicted putative −35 and −10 sequences for each promoter (Fig. S1b). A sequence of approximately 250 bp encompassing −35 and −10 sequences of predicted promoters was cloned in a promoter-less GFP plasmid (pM968) to generate pMp201, pMp622 and pMp922 promoter constructs which were further analysed for GFP expression. Only pMp922 construct was positive for GFP expression, rendering it the most probable promoter for hha and tomB genes (Fig. S2a). This was further validated by the observation that deletion of p922 promoter from the genome resulted in a phenotype exhibited by *hha* and *tomB* deletion mutants (Fig. S2b). Therefore, this promoter was selected for further experiments. Figure S2c shows the nucleotide sequence,−35 and −10 region of p922 promoter sequenced cloned in pM968 plasmid.

**Figure 1:**
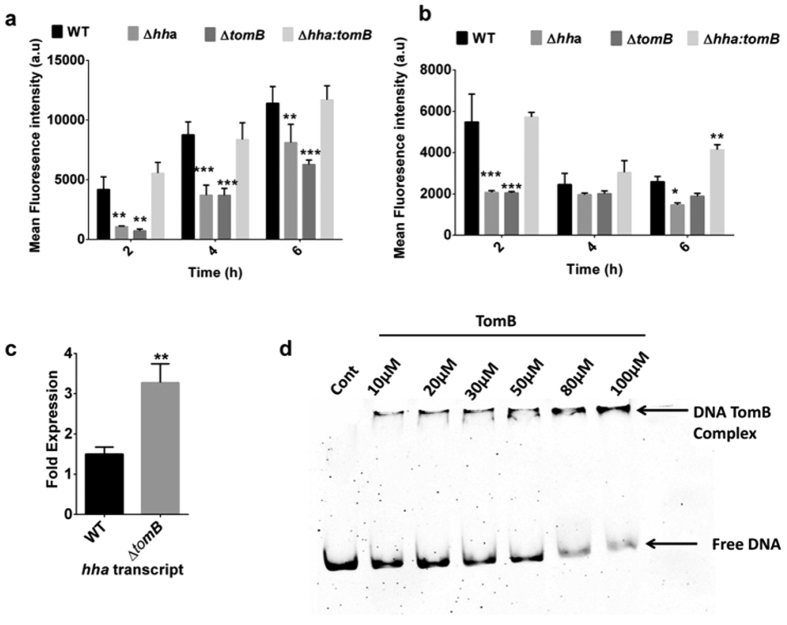
Regulation of p922 promoter by TomB. (**a**,**b**) p922 promoter constructs were transformed into wild-type, ∆*hha*, ∆*tomB* and ∆*hha:tomB*; and the expression of GFP was analysed at indicated time points after growth in LB (**a**) or minimal media (**b**). Reduced GFP expression was observed in ∆*hha* and ∆*tomB* in both media. **(c)** Level of Hha mRNA transcript in wild-type and Δ*tomB.* Overnight grown cultures of wild-type and Δ*tomB* were subcultured and mRNA was isolated with trizol method. Δ*tomB* showed increased mRNA transcript as compared to wild type. **(d)** Electrophoretic mobility shift assay was performed by incubating 100 ng of p922 PCR amplicon with increasing concentration of TomB from 10 μM to 100 μM. A shift in DNA band was observed with increasing concentration of TomB confirming that it binds to p922 promoter.

Next, we investigated the transcriptional regulation of the p922 promoter by TomB. For this, wild-type *S.* Typhimurium, Δ*hha*, Δ*tomB* and double deletion mutant Δ*hha:tomB* harbouring pMp922 GFP constructs were grown in LB or minimal media and GFP expression was analysed by flow cytometry. At the 2 h time point, there was a significant reduction in the GFP expression resulting from the p922 promoter both in LB (p < 0.01) and minimal media (p < 0.001) in Δ*hha* and Δ*tomB,* while GFP expression in the double mutant was comparable to wild type (Fig. 1a and b). A similar trend was observed at 4 h and 6 h in LB, however; in minimal media, at 4 h no significant difference in GFP expression was observed. Additionally, the mRNA transcript levels of *hha* in Δ*tomB* were significantly higher (p < 0.01) than wild-type further confirming the transcriptional regulation of Hha by TomB (Fig. 1c). To validate whether antitoxin mediated repression of p922 promoter was due to direct binding of TomB, an EMSA assay was performed with purified TomB protein and the PCR product of p922. The TomB protein was found to bind to its promoter in a concentration dependent manner (Fig. 1d). The antitoxins of type II TA system have been predicted to bind to palindromic sequences present in the promoter region^1^. An inspection of p922 promoter revealed two palindromes between −10 and start site which can be putative binding sites for TomB (Fig. S2c). In addition transcription of p922 promoter was found to be modulated by growth phase and growth conditions (Fig. S2d and e).

### TomB is labile and forms a stable TA complex with Hha

**Figure 2:**
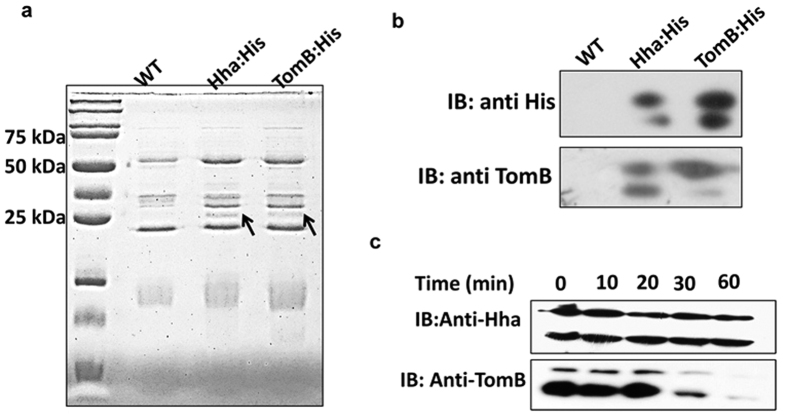
Interaction between Hha and TomB and their stability. (**a**) CBB staining of precipitated proteins. Chromosomal Hha and TomB were tagged with 6X His and precipitated with NiNTA resin. Wild-type lysate was used as control. A band corresponding to a 25 kDa protein was visible in both His tagged protein which was absent in the wild type lysate. **(b)** The lysates of Fig. 2 a were probed with anti His and anti-TomB antibody. His tagged Hha and TomB produced band of similar size. (25 kDa) His tagged Hha precipitated sample also showed band when probed with TomB. **(c)** Pre-existing levels of Hha and TomB were assessed by Western blotting. Wild-type *S.* Typhimurium was grown for 6 hours and treated with 100 μg/ml chloramphenicol. At indicated times samples were harvested and levels of Hha and TomB proteins were determined by Western blotting with anti-Hha and anti-TomB antibody.

To analyse their stability, the levels of pre-existing Hha and TomB proteins were determined by western blotting. TomB showed temporal degradation while the levels of Hha remained same over a 60 min time period (Fig. 2c). This suggests that TomB is labile while Hha is a stable protein. Next, we investigated whether Hha and TomB interact with each other and form a complex. For this, 6X-His tagged Hha and TomB were precipitated with the help of NiNTA resin and analysed by Coomassie staining and Western Blotting. The Hha and TomB His tagged samples showed a band at around 25 kDa which was absent in wild type lysate (Fig. 2a) Interestingly, when probed with anti-His antibody, the Hha and TomB specific bands corresponded to a molecular weight of approximately 25 kDa, which is higher than their actual molecular weight (Fig. 2b). Notably, when the membrane was re-probed with anti-TomB antibody, the 6X-His tagged Hha also showed a band of TomB confirming that TomB was precipitated with Hha. This suggests that Hha and TomB form a stable complex which was not dissociated by denaturing conditions provided during SDS PAGE analysis (Fig. 2b). Moreover, immunoblotting with both anti-His and anti-TomB, two bands were observed suggesting presence of different oligomers.

### The Hha toxin shows conditional toxicity

**Figure 3:**
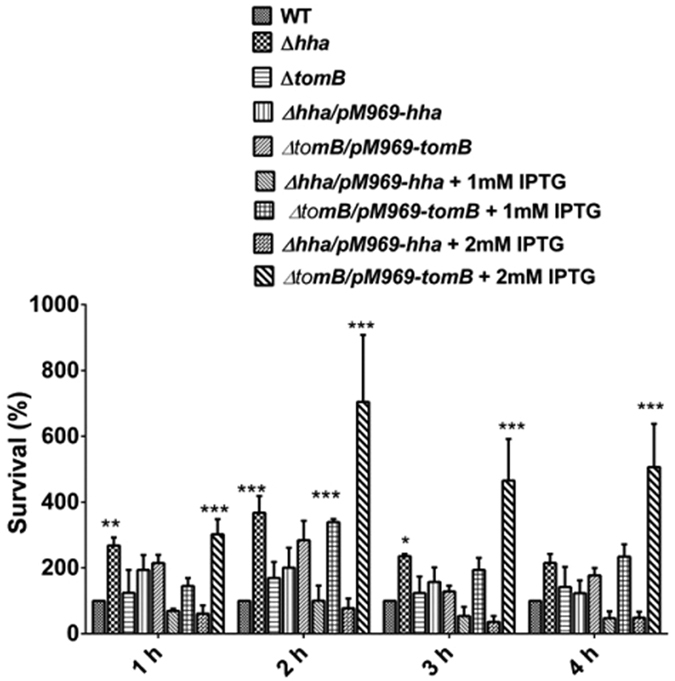
Toxicity of Hha under acid stress. Overnight bacterial cells were subcultured for 2 h at pH 7.5 and subjected to an adaptive pH of pH 4.4 for 1 h. Thereafter, cells were subjected to acid stress of pH 3.1 for indicated times and number of surviving bacteria were enumerated by plating serial dilutions. Error bar indicate the standard deviation of three independent experiments. Level of significance has been indicated by asterisk, *P < 0.05, **P < 0.001, ***P < 0.0001.

To assess the toxicity of the toxin component of Hha-TomB TA module, deletion mutants of respective genes were generated and their growth was compared with the wild type. There was no significant difference in the growth of Δ*hha*, Δ*tomB* or Δ*hha:tomB* in LB or minimal media (Fig. S3a and d). When *hha* and *tomB* genes, under an inducible promoter, were complemented in their respective deletion mutants and over expressed, no significant difference in growth in either LB or minimal media was observed (Fig. S3b and c). Various TA systems have been shown to be activated by stress conditions. Therefore, we compared the survival of Δ*hha* and Δ*tomB* with wild-type *S*. Typhimurium under acid stress. At 1 h post challenge, deletion of the *hha* toxin gene resulted in an almost 3 fold greater survival (p < 0.05) as compared to the wild-type strain (Fig. 3). Similar trend was observed at later time points. Further, overexpression of *hha* decreased survival under acid challenge as compared to the wild-type. However, the difference was not statistically significant. Although deletion of *tomB* did not affect its survival under acid stress, its overexpression increased the survival fitness of *S.* Typhimurium by 4–6 folds as compared to the wild-type strain (Fig. 3). Significant increase (p < 0.001) in the survival of the strain overexpressing *tomB* was observed at all-time points. Taken together, these data suggest that the toxicity of Hha is induced by acid stress.

### Hha-TomB TA system is involved in persister cell formation

**Figure 4:**
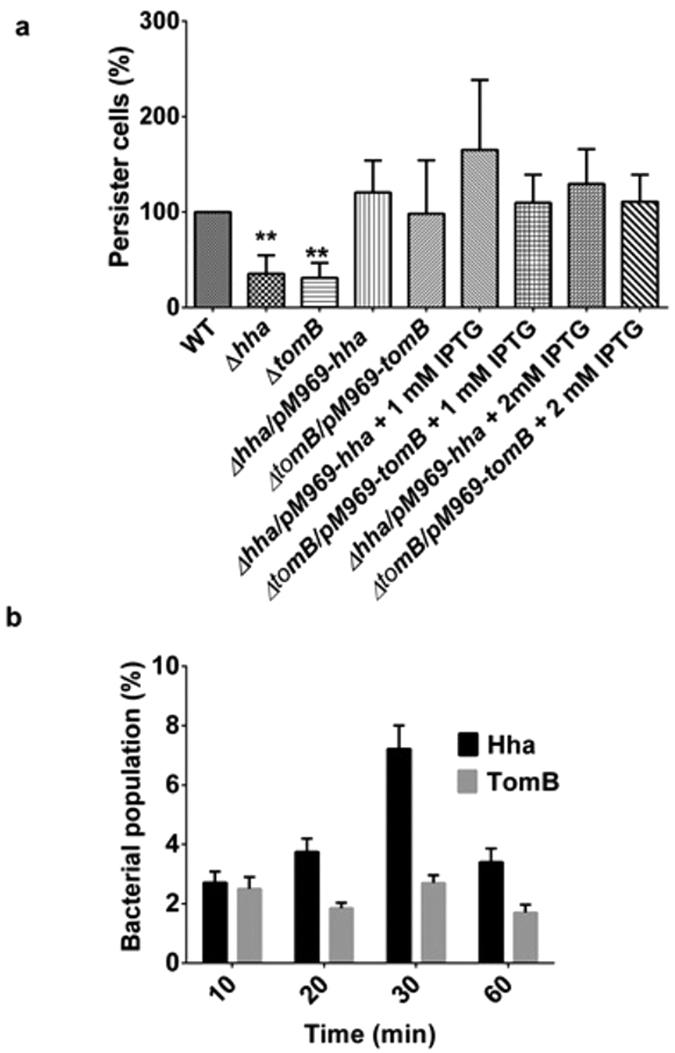
Persistence in WT, ∆*hha* and ∆*tomB* mutant and complemented strains. (**a)** Wild-type *S.* Typhimurium, mutants and complemented strains were grown overnight in LB supplemented with appropriate concentrations of antibiotics and IPTG. Overnight grown bacterial cells were subcultured for 2 h and gentamicin 10 μg/ml was added followed by incubation for 2 h. The numbers of persister cells were enumerated by plating serial dilutions. Level of significance has been indicated by asterisk, *P < 0.05, **P < 0.001, ***P < 0.0001. **(b)** Antibiotic treated bacterial cells were immunostained with respective primary and FITC conjugated secondary antibody and analysed on flow cytometry. The indicated figure shows the bacterial population percentage having fluorescence intensity that exceeds the maximum intensity shown by untreated cells. The experiments were performed three times.

**Figure 5:**
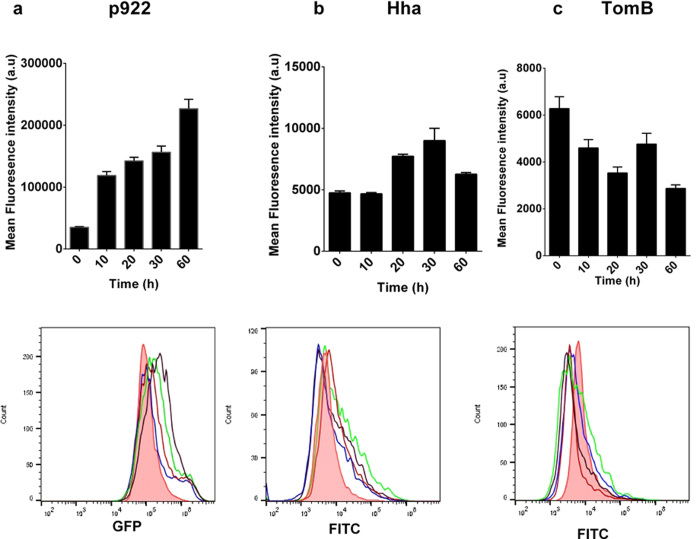
Transcription of p922 and expression of Hha and TomB after gentamicin treatment. (**a**) GFP reporter assay: Wild type *S.* Typhimurium harbouring p922 GFP construct was grown overnight, subcultured for 2 h and treated with gentamicin. 1 ml of culture was withdrawn at indicated time points and analysed on flow cytometer for level of GFP expression. (**b**,**c**) Immunostaining: Overnight bacterial cells were subcultured for 2 h and treated with gentamicin (100 μg/ml). 1 ml of culture was withdrawn at indicated time points and immunostaining was performed with FITC-conjugated secondary antibody. Fluorescence was measured by flow cytometry. Pink (Filled): 0 min, Blue: 10 min, Orange: 20 min, Green: 30 min, Black: 60 min.

Hha has been shown to be required for persister cell formation in *E. coli*^20^. Therefore, we investigated for the same role in *S.* Typhimurium. Persister cell formation by Δ*hha* and Δ*tomB* was compared with wild-type after treatment with gentamicin as described in materials and methods. Almost 50% reduction in persister cell formation was observed in both deletion mutants (Fig. 4a). Complementation of deletion mutants with respective genes and overexpression with IPTG resulted in restoration of persister phenotype (Fig. 4a). To find relation between Hha/TomB expression and persistence, we analysed population level expression of Hha and TomB after gentamicin treatment. While untreated cells showed uniform population, soon after antibiotic treatment, a heterogeneous population of bacterial cells were present expressing different levels of Hha and TomB (Fig. 5). Between 20 to 60 min post treatments mean fluorescence intensity for Hha expression was higher than untreated cells (Fig. 5a). However, a subset of bacterial population (2–7%) existed whose fluorescence intensity exceeded the maximum fluorescence shown by untreated cells (Fig. 4b). Similarly, the TomB expression went down in most cells after treatment, however, a sub-population existed which had higher TomB expression level (Fig. 5c). In order to corroborate our finding, we also measured the transcription of p922 promoter by GFP reporter assay and found that p922 transcription increased after the treatment. Similar to Hha and TomB expression, the transcription of p922 was found to be heterogeneous at population level with a sub-population showing one log increased transcription as compared to untreated cells (Fig. 5a).

### The Hha-TomB TA system plays an important role in antibiotic induced programmed cell death

**Figure 6:**
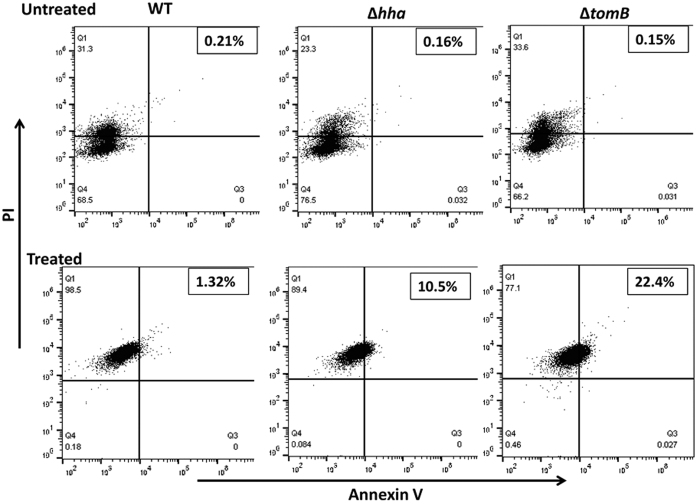
Annexin V labelling. Overnight bacterial cells were subcultured for 2 h and treated with gentamicin (100 μg/ml). 1 ml of culture was withdrawn at 4 h post treatment and stained with FITC-conjugated Annexin V. Fluorescence was measured by flow cytometry.

Various studies have reported the involvement of Bacterial TA systems in programmed cell death. The *E. coli,* MazEF TA pair mediate stress induced programmed cell death^22^ but *S.* Typhimurium lacks MazEF TA system^23^. We investigated whether Hha-TomB has any role in PCD. Similar to *E. coli*^24^, *S.* Typhimurium was also found to exhibit hallmarks of apoptosis upon antibiotic treatment (Figs 6, S4 and S5). To investigate the involvement of Hha-TomB in antibiotic induced PCD in *S.* Typhimurium, hallmarks of apoptosis were compared in wild-type and mutants. As described in materials and methods, the bacterial cultures were treated with gentamicin and the number of viable cells was determined at indicated time points post treatment. A sharp decline in viability was observed between 1 and 2 h of the antibiotic exposure followed by a gradual decrease in the wild type and the mutants of hha and tomB. However, this was more coherent in Δ*hha* and Δ*tomB* as compared to wild-type (S4a).

Phosatidylserine (PS) flipping to the outer membrane is an important marker for apoptosis. Therefore, we investigated exposure of PS by the binding of FITC labelled Annexin V. Percentage of AnnexinV positive cells was higher in Δ*hha* (10.5%) and Δ*tomB* (22.4%) as compared to wild type (1.32%) (Fig. 6). This finding suggests that Hha and TomB when present together in wild-type inhibit apoptosis-like death (ALD) while absence of either of the gene is unable to exert such inhibition.

A very similar observation was made in case of DNA fragmentation which was assessed by TUNEL assay. In wild-type 4.21% cells were found to be TUNEL positive which increased to 12.8% in Δ*hha* and 15.8% in Δ*tomB* (Fig. S4b).

Besides, PS exposure and DNA fragmentation, chromosomal condensation is also one of the apoptotic markers. In order to analyse the changes in the structure of the chromosome upon gentamicin treatment, bacterial cells were stained with DNA conformation sensitive dye Hoechst 33258 and imaged with fluorescence microscope. There was a marked difference in the chromosomal staining pattern of wild-type and mutants. Wild-type bacterial cells showed intense fluorescence covering the entire length of bacterial cells. On the other hand, in mutants the staining was dim and focused (Fig. S5). These findings suggest a substantial difference in conformation of the chromosome of mutants and wild type

**Figure 7:**
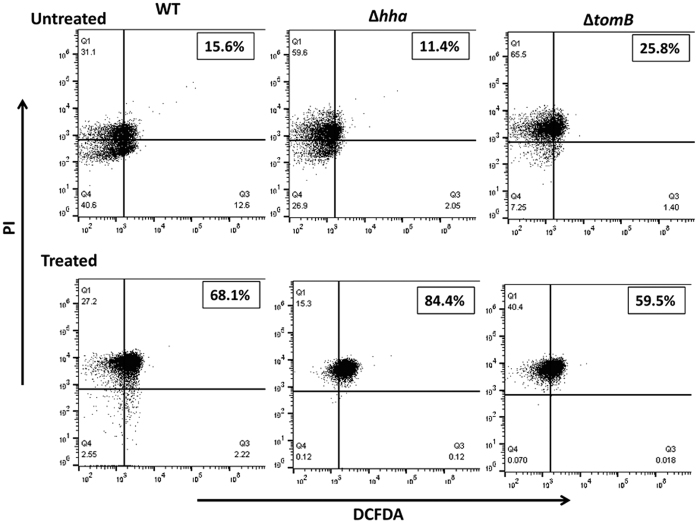
ROS production. Overnight grown bacterial cells were subcultured for 2 h and treated with gentamicin (100 μg/ml). 1 ml of culture was withdrawn at 4 h post treatment and DCFDA dye (10 μM) was added. Fluorescence after 30 min was measured by flow cytometry. PI was used as counter stain

Reactive oxygen species (ROS) are one of the potent inducers of PCD. Therefore, we assessed whether, in present case, ALD was accompanied by generation of ROS. At first, generation of ROS was studied in wild-type *S.* Typhimurium after treatment with gentamicin. ROS production increased temporally with almost 94% ROS positive cells after 4 h of gentamicin treatment (Fig. S6). Therefore, ROS generation in WT and mutants was compared at 4 h post antibiotic stress. It was observed that wild-type and mutants showed increased number of ROS producing cells in treated population as compared to untreated ones with highest ROS generation in Δ*hha* (84.4%) followed by to wild-type (68.1%) and Δ*tomB* (59.5%) (Fig. 7). Notably, despite increased number of apoptotic cells in Δ*tomB,* number of ROS producing cells was considerably lower than wild-type. Thus, it is evident that, in present case, ALD was not triggered by ROS. Taken together, these findings confirm that deletion of *hha* and *tomB* increases the number of apoptotic cells providing evidence for the involvement of Hha-TomB TA system in antibiotic induced programmed cell death.

## Discussion

The Hha protein belongs to the YmoA family of regulators which is known to be involved in the regulation of virulence factors in enteropathogenic bacteria like *E. coli* and *Salmonella*^8^. It is a global transcriptional regulator and controls the expression of several environmentally regulated genes. It is reported to be a modulator of environmental stimuli such as osmolaority and temperature^25^ and causes pleotropic effects by modulating multiple genes^26^. By interacting with the H-NS protein, it plays an important role in motility and survival under low oxygen conditions^21^. In *E. coli*, Hha has been identified as a thermo and osmo-modulator. In *E. coli, hha* has been proposed to form a TA system with its adjacent gene *tomB*^27^. However, very limited literature is available on TomB of *E. coli.* It has been annotated as an uncharacterized hypothetical protein in *Salmonella*. Despite a number of studies on the structure and function of Hha in *E. coli* and *Salmonella*, its detailed characterization as a TA module is lacking. In this study, we have performed extensive characterization of the Hha-TomB as a TA module and examined their role in *S.* Typhimurium.

Type II TA modules exhibits a set of characteristic features which include: (1) genes for toxin and antitoxin present in the same operon, (2) the antitoxin binds directly with the toxin and forms a stable complex, (3) the antitoxin is labile while toxin is comparatively stable and (4) the antitoxin regulates transcription of its own promoter. In the present study, Hha and TomB satisfy all of the above criteria. The antitoxin TomB was found to repress the transcription of the p922 promoter by direct binding. In addition, two palindromic sequences between −10 and the transcriptional start site were identified, which serve as putative binding sites for the antitoxin. It was observed that, TomB was co-precipitated with Hha confirming complex formation through direct binding, an association that was not sensitive to denaturing environments provided during SDS PAGE. Furthermore, two different oligomers were visible in western blots. The crystal structures of several TA complexes have shown that toxin and antitoxin monomers form oligomers in a stoichiometric manner^28^. A study on the structure of BrnTA toxin antitoxin module showed that the toxin BrnT formed unusual higher order oligomers under denaturing condition which was visible on coomassie stained SDS PAGE gel as a ladder of protein bands^29^. The authors could not explain the reason for the formation of such structures under denaturing conditions^29^. Therefore, it may be possible that Hha and TomB form different oligomers that remain stable under denaturing conditions. Based on the evidences cited above, we describe Hha and TomB as a new type II TA module of *S.* Typhimurium.

The expression of Hha-TomB TA system was modulated by growth environment as the promoter of this TA system showed increased expression with time in LB while in minimal media opposite was observed. LB and minimal media differ in their nutrient content. LB is nutrient rich while minimal media presents stress. It might be possible that long term exposure to nutrient stress represses the transcription from p922 promoter to minimise the actions of this TA system.

Generally, deletion of antitoxin increases the level of free toxin which exerts its toxicity resulting in cell death or growth arrest. However, in the present study, deletion of neither toxin nor antitoxin had any effect on the growth of *S.* Typhimurium in liquid LB or minimal media. Our finding was in line with previous reports where deletion of Hha or TomB (named as YbaJ) did not affect the growth in minimal or complex media in relation to wild type^30^. On the other hand in biofilm, Hha was found to cause cell death while TomB attenuated its toxicity^27^. Notably, neither deletion of antitoxin nor overexpression of toxin showed any bacteriostatic or bactericidal effect in the above media. However, under acid stress, same was found to be toxic. Further, overexpression of cognate antitoxin also provided survival fitness under acid stress condition. Thus based on the present study and previous finding^27^ we conclude that Hha toxicity is conditional and is induced by certain stimuli such as acid stress or biofilm formation. The mechanism of regulation of Hha toxicity is not known. However, it is possible that being an environmental-response regulator, in response to certain environmental stimuli, it induces genes which cause cell lysis. One mechanism through which it can cause toxicity is by activation of phage lytic genes. In *E. coli*, Hha was shown to cause toxicity in biofilms by activating prophage lytic genes^27^. Moreover, overexpression of Hha also induces ClpP, ClpX and Lon proteases which activate other toxins by degrading their cognate antitoxin^27^. A similar mechanism may operate in *S.* Typhimurium. In the data set reported by Ryan *et al*.^31^, Hha is upregulated almost 6 folds under acid challenge^31^. Furthermore, Hha over expression was accompanied by upregulation of most of the proteases such as ClpA, ClpP, ClpX and Lon as well as phage proteins^31^. Thus it is apparent that similar to biofilms, Hha expression is upregulated under acid stress resulting in the activation of proteases and phage proteins, causing cell lysis.

**Figure 8:**
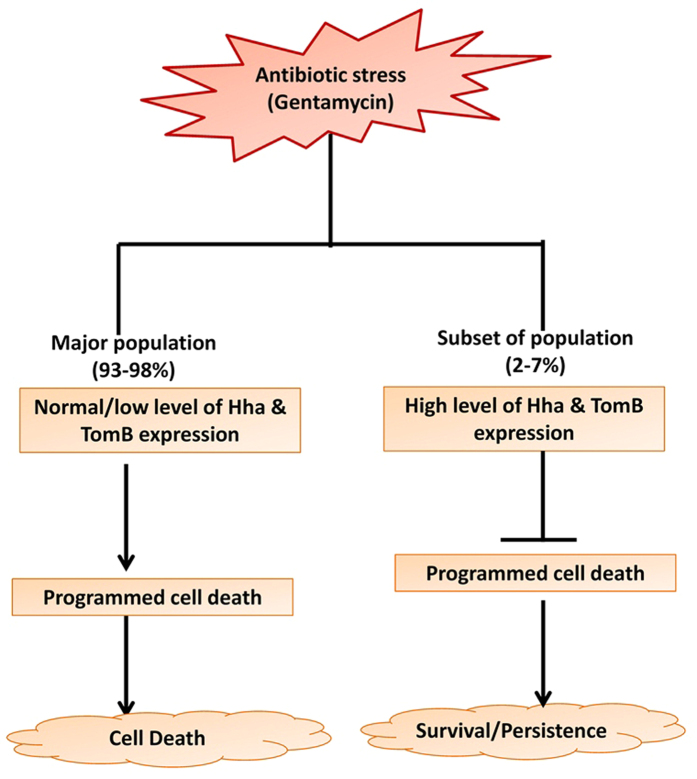
Proposed model for Hha-TomB TA system mediated persistence. Under antibiotic stress, subsets of bacterial population increases expression of Hha-TomB TA system which in turn inhibit programmed cell death and prevent bacteria from committing suicide. Surviving bacteria form persister cells. Population express normal to low level of Hha and TomB and undergo apoptosis following antibiotic treatment resulting death of most of the population.

Conditional toxicity of Hha is also evident from the finding that under antibiotic stress, rather than causing cell death Hha promoted persister cell formation. Similar to Hha, TomB was also found to promote persister cell formation, since deletion of both the genes resulted in decreased persistence. When bacterial populations are subjected to lethal stress, few cells adjust their metabolism so that they can withstand the lethal effects of the stress. Similar observations were made in the present study where, heterogeneous population of bacteria expressing different levels of Hha and TomB were observed post antibiotic treatment. Interestingly, this TA system was found to inhibit antibiotic induced PCD. Inhibition of PCD could be one of the mechanisms of persister cell formation. It was observed that post gentamicin treatment few bacteria increased Hha and TomB expression. Since deletion of both genes resulted in the increased number of apoptotic cells, therefore, it can be assumed that few bacteria expressing high levels of Hha and TomB would survive the lethal action of antibiotic due to inhibition of intracellular death programs leading to formation of persister cells. Therefore we propose that inhibition of PCD is one of the mechanisms by which the Hha-TomB TA system promotes persister cell formation (Fig. 8).

ROS are known to be potent inducers of PCD^32^. Generally, endogenous oxidant remediation systems remove ROS produced by cells^33^, however, when ROS generation exceeds the level manageable by above system, then ROS can trigger intracellular death^34^. In the present study ALD was not directly associated with ROS generation because Δ*tomB* showed the highest number of apoptotic cells but lowest number of ROS positive cells. Therefore, gentamicin treatment indeed induced ROS production; however, it did not trigger ALD in present case.

Till date, Hha had been represented as a modulator of virulence in *Salmonella.* In this study, a new function of Hha as a toxin was described and its role in persistence and PCD was elucidated. This is the first report of any nucleoid associated protein functioning as a TA module in *Salmonella*. In addition, the present study provides evidence for TomB as an Hha toxicity attenuator under acid stress in *S.* Typhimurium.

## Materials and Methods

### Bacterial strain and growth condition

The bacterial strains and plasmids used in this study have been listed in Tables 1 and 2 respectively. Wild type *S.* Typhimurium SB300^35^ and its isogenic mutants were grown in Luria Bertani (LB) medium (10 g/L Bacto tryptone, 5 g/L Bacto yeast extract, 5 g/L NaCl, HiMedia, India) or minimal E glucose medium (minimal EG) containing MgSO_4_.7H_2_O (200 mg), citric acid.H_2_O (2 g), K_2_HPO_4_ (10 g), NaH_2_PO_4_ (1.75 g), (NH_4_)_2_HPO_4_ (1.75 g) in 1 L of Milli-Q water, adjusted to pH 7.5^36^. The minimal medium was supplemented with 0.4% dextrose and 0.1% casein hydrolysate (both 0.22 μm filtered). Antibiotics in growth media were used at the following concentrations: ampicillin 100 μg/ml, kanamycin 50 μg/ml, streptomycin 50 μg/ml and chloramphenicol 20 μg/ml. Bacterial strains were grown at 37 °C in a shaking incubator at 150 rpm.

### *In silico* promoter identification

The gene sequences of *hha* and *tomB* as well as their organization on the chromosome of *S.* Typhimurium SL3144 were obtained from NCBI (NC_016810.1). For identifying the promoter, a sequence 1000 bp upstream of *tomB* was retrieved and analysed by BPROM software maintained by Softberry (http://www.softberry.com/berry.phtml?topic=bprom&group=programs&subgroup=gfindb)^37^. Predicted promoters were cloned into a promoterless GFP plasmid pM968^38^ between XbaI and BamHI restriction sites to create the constructs pMp201, pMp622 and pMp922.

### Generation of deletion mutants and complementation

Gene deletion was performed using one-step inactivation method^39^. Briefly, the *hha* and *tomB* genes were replaced with Tn5 neomycin phosphotransferase gene (aphT) (conferring resistance to kanamycin) by a previously described protocol^40^. For complementation, the *lac* promoter along with its operator was cloned in pM968 plasmid to generate pM989 and the GFP coding sequence was replaced with *hha* or *tomB* genes. All primers have been listed in Table 3.

### Validation of promoter activity through GFP reporter assay

The three promoter constructs mentioned above were tested for GFP expression with the positive one (pMp922) being transformed into WT, *Δhha,* Δ*tomB* and Δ*hha:tomB* strains. These transformants, with the pM922 construct were grown in LB or minimal media and GFP expression was analysed by the flow cytometry. The experiment was performed in biological triplicate.

### Cloning, expression and purification of recombinant Hha and TomB proteins

For cloning, *hha* and *tomB* genes were amplified from genomic DNA of wild-type *S.* Typhimurium SB300 and cloned into pET28a vector between NdeI and XhoI sites so as to have an N-terminal 6X His tag. The recombinant pET28a::*hha* and pET28a::*tomB* constructs were transformed into *E. coli* BL21 (DE3) CodonPlus cells and the transformants were selected on Luria-Bertani (LB) agar media supplemented with kanamycin (50 μg/ml). Transformed colonies were inoculated in liquid culture and incubated at 37 °C and 150 rpm until an OD_600_ of 0.6. At this stage 1% of the primary culture was subcultured in 2 litres LB media supplemented with kanamycin (50 μg/ml). When the OD_600_ reached 0.6–0.8, cells were induced with Isopropyl 1-thio-β-D-galactopyranoside (IPTG) to a final concentration of 1 mM and kept in the shaker incubator at 16 °C for 16 h. Cells were then harvested by centrifugation at 4000 g for 20 min and resuspended in lysis buffer (50 mM Tris pH 8.0, 50 mM NaCl, 10 mM imidazole and 2 mM β-Me) containing protease inhibitor cocktail (Roche). Lysis was done by sonication and the lysate was centrifuged at 13000 g for 45 min to remove cell debris. Ni^2+^ resin (Qiagen) was equilibrated in the same cell lysis buffer and the lysate was incubated with the resin at 4 °C for 30 min on a rotator. The cell lysate and Ni^2+^ resin mixture was then subjected to affinity-based gravity-flow column chromatography after being washed with a buffer containing 50 mM Tris pH 8.0, 50 mM NaCl, 30 mM imidazole and 2 mM β-Me. The recombinant protein was eluted with increasing concentrations of imidazole in the above mentioned buffer. Fractions were further analyzed in 15% SDS-PAGE. Eluted fractions were pooled and concentrated using a 3 kDa molecular-weight cut off centrifugal filters (Millipore). The concentrated protein samples were further purified using Sephadex-75 gel filtration chromatography (GE Healthcare) which was pre-equilibrated with a buffer (Hha: 20 mM phosphate buffer pH 6.5, 50 mM NaCl and 2 mM β-Me and TomB: 20 mM phosphate buffer pH7.0, 200 mM NaCl, 2 mM β-Me). The fractions were subsequently analyzed by SDS-PAGE. The protein fractions were pooled and dialysed with phosphate buffer saline (PBS) pH 7.4. The 6X-histidine residues were removed by thrombin cleavage and the protein was purified through benzamidine column (GE Healthcare) according to manufacturer’s protocol.

### Antibody generation and characterization

Polyclonal antibody against Hha and TomB was raised in rabbit. New Zealand White rabbits were purchased and maintained in the animal house facility of KIIT School of Biotechnology, Odisha, India. For generation of antibody, approval from the Institutional Animal Ethics Committee (IAEC), KIIT University was obtained. A previously published method was used for immunization and serum isolation^41^. Briefly, 10 weeks old rabbits were immunized with 0.5 mg respective proteins (purified by above method) in complete Freund’s adjuvant in 1:1 ratio followed by booster doses (0.25 mg antigen in incomplete Freund’s adjuvant in 1:1 ratio) at 14^th^, 28^th^ and 42^nd^ day of immunization. Serum was collected on 35^th^ and 56^th^ day with antibody titres being determined by Enzyme Linked Immunosorbent Assay. Whole serum was divided into small aliquots and stored at −80 °C. The Antibody thus generated was used in western blot experiments.

### Electrophoretic mobility shift assay (EMSA)

EMSA experiments were performed as described previously^8^ with some modification. 100 ng of the PCR product corresponding to the p922 promoter region was incubated with increasing concentrations (10–100 μM) of TomB in 50% glycerol for 1 hour at 25 °C followed by further incubation with antibody specific to TomB (1:1000) for 1 hour under similar conditions. The reaction mixture was separated by electrophoresis in 10% polyacrylamide gels and Tris-borate EDTA buffer. The DNA bands were visualized by staining with ethidium bromide. The experiment was performed biological triplicate.

### Protein stability analysis of Hha and TomB

The stability of toxin and antitoxin was determined by a previously described procedure^8^. *S.* Typhimurium SB300 was grown for 6 h in LB medium. Protein synthesis was stopped by the addition of 100 μg/ml chloramphenicol, and samples were removed at the indicated time points. The level of Hha and TomB at each time point was determined by Western Blotting using a polyclonal anti-Hha and anti-TomB antibody.

### Study of Interaction between Hha and TomB

Analysis of the interaction between Hha and TomB was performed by a previously described protocol^42^. Briefly, cell lysates expressing chromosomally His-tagged Hha and TomB were allowed to bind to Ni^2+^NTA beads for precipitation of His tagged protein. The bound beads were washed with 50 mM Tris pH 8.0 and 10 mM imidazole to remove any non-specific binding. The target proteins were eluted out against the competitive binding of imidazole (200 mM). Eluents were separated on a 12% SDS-PAGE gel and transferred to a PVDF membrane. Detection of His tagged proteins was done against anti-His Antibody. Confirmation of the interaction was done by detecting the same bands with anti-TomB antibody.

### RNA isolation and qRT PCR

For RNA isolation, wild-type *S.* Typhimurium and mutants were grown overnight in LB, and subcultured for 4 h. Total RNA was extracted from 1 ml of bacterial culture grown under the afore mentioned conditions, using Trizol reagent (Ambion, USA) as per the manufacturer’s instructions. Subsequently, the samples were treated with RNase free DNase I (Fermentas) followed by cDNA synthesis using the Hi-cDNA Synthesis Kit (HIMEDIA, India). At each stage the quantity and purity of RNA was determined by Epoch Microplate Spectrophotometer (BioTek, USA). qPCR was carried out for each sample using the KAPA SYBR FAST qPCR Master Mix (2x) (KAPABIOSYSTEMS, USA) with a suitable cDNA dilution as template. Presence of genomic DNA (gDNA) contamination was checked by running DNase treated RNA as a control. To normalize the expression of the tested genes, gmk (guanylate monophosphate kinase) was used as an internal control. Mean fold change expression values were determined for three biological and technical replicates.

### Analysis of growth

The wild-type, mutants and complemented strains were grown overnight in LB media or minimal media supplemented with appropriate antibiotics at 37 °C, under shaking condition at 150 rpm. 1% of the overnight culture was inoculated into fresh LB or minimal media and incubated at 37 °C, 150 rpm. For analysis of growth, 1 ml of culture was withdrawn at indicated time points and number of colony forming unit (cfu) was determined by serial dilution and plating. The experiment was performed in biological triplicate and area under curve (AUC) analysis was performed to determine differences in growth kinetics.

### Survival under acid stress

Survival under acid stress was analysed by a slightly modified procedure described previously^36^. Briefly, bacterial strains were grown overnight in minimal media (pH7.5) supplemented with appropriate antibiotics at 37 °C. The overnight culture was diluted with fresh minimal media in 1:200 ratios and allowed to grow under similar conditions. After 2 hours, growth media was replaced with fresh minimal media at pH 4.4. Following 1 h incubation at pH 4.4, cells were acid stressed by replacing the media with fresh minimal media having pH 3.1. The number of surviving bacteria pre and post acid stress was determined by serial dilution and plating. The survival percentage was determined by the ratio of post acid stress cfu to pre-acid stress cfu. Means were evaluated with Two-way ANOVA and Bonferroni’s multiple comparison was performed using wild-type as control. The experiment was performed three times.

### Analysis of persistence

Persister cell formation was analysed by a previously published protocol with some modifications^20^. Wild-type, isogenic mutants and complemented strains were grown overnight in LB media and sub-cultured (1: 200 ratio) for 2 h under similar conditions followed by treatment with gentamicin10 μg/mL for 2 h. For analysis of persistence, 100 μl of the culture was withdrawn at pre and post antibiotic treatment and surviving cfu was determined by serial dilution and plating. The persistence was expressed as a percentage of surviving bacteria post-treatment versus pre-treatment. The Experiment was performed in biological triplicate.

### Analysis of expression of Hha and TomB by flow cytometry

*S.* Typhimurium was grown and treated with gentamicin as mentioned above and 1 ml of samples were withdrawn at 0, 10, 20, 30 and 60 min. Cells were fixed with 4% paraformaldehyde and permeabilised with triton X-100. Cells were blocked with 5% BSA for 30 min. Primary antibodies were added at 1:100 dilution and FITC conjugated secondary antibody was added at 1:1000 dilutions. Cells were washed and analysed on flow cytometer (Attune Acoustic Focusing cytometer, Applied Biosystems). The experiment was performed in biological triplicate.

### Western blot analysis

Cell lysates from different experiments were prepared by sonication, quantified by Bradford method and 100 μg of total protein was run on SDS PAGE gels. The protein samples were electro-transferred to Polyvinylidenefluoride membrane and blocked with 5% bovine serum albumin in TBST (20 mM Tris-HCl [pH 7.6], 0.15 M sodium chloride, and 0.5% Tween 20), for 2 h at room temperature, followed by incubation with primary antibody in TBST overnight at 4 °C. The membrane was washed three times with TBST and incubated with horseradish peroxidase-conjugated anti-mouse or -rabbit immunoglobulin for 2 h. After three washes with TBST, the blot was developed with the enhanced chemi-luminescence system (Abcam, UK) according to the manufacturer’s instructions.

### Analysis of antibiotic induced programmed cell death

Programmed cell death was analysed according to the method described by Dwyer, *et al*.^24^, with some modification^24^. The Overnight cultures of the wild-type and mutant strains were sub-cultured in a 1:200 ratio for 2 h at 37 °C, followed by treatment with 100 μg/mL gentamicin and subsequent incubation with antibiotics for 4 h. The percentage of viable cells at 1 h, 1.5 h, 3 h and 4 h was determined by plating serial dilutions. For detecting the hallmarks of apoptosis, wild type and mutants were treated with gentamicin and incubated for 4 h as mentioned above. The quantification of the Reactive Oxygen Species (ROS) was performed by the protocol described by Li and colleagues with some modification^43^. 2′,7′-dichlorofluorescindiacetate (DCFDA) fluorescent dye was added to the culture at 10 μM concentration during treatment with gentamicin and mean fluorescence intensity was determined after 30 min by flow cytometry (Attune Acoustic Focusing cytometer, Applied Biosystems) at indicated time points. Propidium Iodide (PI) was used as counterstain.

Phophatidylserine exposure was measured by Annexin V-FITC detection Kit (Sigma). Cells were stained with FITC conjugated Annexin V according to manufacturer’s protocol and analysed by flow cytometry (Attune Acoustic Focusing cytometer, Applied Biosystems).

For analysis of DNA structure, cells were grown and treated with gentamicin as described above. After 4 h of antibiotic treatment, Hoechst 33258 (PolysciencesInc, US) was added at the concentration of 10 μg/ml and incubated on ice for 30 min. Counterstaining was done with Propidium Iodide and cells were analysed with a fluorescence microscope (Olympus BX61, ImagePro ExpressTM). Images were processed by ImageJ.

For analysis of DNA fragmentation, TUNEL assay was performed using *In Situ* Direct DNA Fragmentation Assay Kit (Abcam; ab66108, UK) according to the manufacturer’s protocol. The FITC labelled cells were analysed by Flow cytometry (Attune Acoustic Focusing cytometer, Applied Biosystems). All the PCD experiments were performed in biological triplicate.

### Statistical analysis

All the *in vitro* data represent mean ± standard deviation of three independent experiments. One and Two-way Analysis of Variance (ANOVA) was employed to determine significant differences wherever applicable. All the statistical calculations were performed with the help of GraphPad Prism software version 6.

### Ethics statement

Immunization experiments were performed according to the guidelines of the Institutional Animal Ethics Committee (IAEC), (Registration number: 1577/PO/Re/S/2011/CPCSEA) KIIT University; which follows the guidelines and protocols laid by Committee for the Purpose of Control and Supervision of Experiments on Animals (CPCSEA), Ministry of Environment, Forests and Climate Change. Antibody generation was carried out under the approval number: KSBT/IAEC/2014/MEET-1/A6 obtained from IAEC, KIIT University.

## Additional Information

**How to cite this article**: Jaiswal, S. *et al*. The Hha-TomB Toxin-Antitoxin System Shows Conditional Toxicity and Promotes Persister Cell Formation by Inhibiting Apoptosis-Like Death in *S.* Typhimurium. *Sci. Rep.* **6**, 38204; doi: 10.1038/srep38204 (2016).

**Publisher's note:** Springer Nature remains neutral with regard to jurisdictional claims in published maps and institutional affiliations.

## Supplementary Material

Supplementary Information

## Figures and Tables

**Table 1:** Bacterial strains used in this study.

Bacterial strain	Genotype and/ or relevant characteristics	Resistance	Source or reference
Wild-Type (WT)	*Salmonella* Typhimurium SB300,	*Sm*^*r*^	35
Δ*hha*	*hha::aphT*	*Sm*^*r,*^*Km*^*r*^	This study^35^
Δ*tomB*	*tomB:aphT*	*Sm*^*r,*^*Km*^*r*^	This study
Δ*hha:tomB*	*hha::aphT, tomB::cat*	*Sm*^*r,*^*Km*^*r*^*, Cm*^*r*^	This study
Δ*hha/p969-hha*	Δ*hha* complemented with *hha* gene through pM969 plasmid	*Sm*^*r,*^*Km*^*r*^*, Amp*^*r*^	This Study
*ΔtomB/pM969-tomB*	ΔtomBcomplemented with *tomB* gene through pM969 plasmid	*Sm*^*r,*^*Km*^*r*^*, Amp*^*r*^	This Study

**Table 2:** Plasmids used in this study.

Plasmids	Genotype and/ or relevant characteristics	Resistance	Source or reference
pM986	Promoterless GFPmut2 in promoterless pBAD24 vector	*Amp*^*r*^	38
pM969	Lac promoter cloned in pM968 between BamHI and XbaI	*Amp*^*r*^	This study
pMp922	922 promoter cloned in pM968 between BamHI and XbaI	*Amp*^*r*^	This study
pMp622	622 promoter cloned in pM968 between BamHI and XbaI	*Amp*^*r*^	This study
pMp201	201 promoter cloned in pM968 between BamHI and XbaI	*Amp*^*r*^	This Study
pET28a	T7 promoter containing plasmid with pBR322 origin	Km^r^	Novagen, USA
pKD4	Template plasmid; FRT-*aphT*-FRT		39
pKD46	Red recombinase expression plasmid; P_araB_; oriR101		39

**Table 3:** Primers used in this study.

Primers	Sequence (5′ to 3′)
Fwkohha	GAGGCAGATAACACCTGCGTGTTCTCTAAAAAGTAATGTAGCGTGATGTGTAGGCTGGAGCTGCTT
Rwkohha	GTTAGTTTGTCTTGTTAAAAATTATTACAATCATAGGTAGAATTTATATGAATATCCTCCTTAGT
Confhha	TCGTCACTC ATCGCGCGCT C
FwkotomB	TAATGGTTTATCAGACATAAATTCTACCTATGATTGTAATAATTTTGTGTAAGGCTGGAGCTGCTT
RwkotomB	CCGAAGGTGTTCGGTTAGTTTAAGCCACTAAAAAGGGGATGCATTATATGAATATCCTCCTTAGT
ConftomB	GCGCCGTAAACGCATCAAAT
FwLacpM968	GGCCGCTCTAGAACGACAGGTTTCCCGACTGG
RwLacpM968	GGCCGTGGATCC AGCTGTTTCCTGTGTGAAATTG
FwhhapM969Pst1	GGCCGCTA CTGCAG ATGTCTGATA AACCATTAAC
RwHhapM969HindIII	GGCCGCTA AAGCTT TTAACGAATGAATTTCCATAC
FwTomBM969BamHI	CGCGGATCCATGGACGAATACTCAC
RwTomBpM969HindIII:	GGCCGCTA AAGCTT TTAACAAGACAAACTAAC
FwP922M968 XbaI	GGCCGC TCTAGA AACTGGTTCCTTTTTAGGCG
RwP922M968BamH1	GGCCGT GGATCC AATGCATCCCCTTTTTAGTG
FwP622M968 XbaI	GGCCGC TCTAGA TACTGGGCGGGATGGTAACG
RwP622BamH1	GGCCGT GGATCC ATTGTTTGTGTAATCATTGG
FwP201M968 XbaI	GGCCGC TCTAGA TCGTCGTCTTCCTGTGTCTG
RwP201M968BamH1	GGCCGT GGATCC CGCCAGTACCTACCGCATTC
FwKoP922	ACACGCGGCTTTCACGCAATTGTAATTTTTAGTAATATGACGATGGTGTAGGCTGGAGCTGCTTC
RwKoP922	CCTTCGGGATGGATACAACTAACTAACCTGGTACTTCCCACGTCACATATGAATATCCTCCTTAG
FwhhapET28aNde1	GGCCGCTA CATATG TCTGATA AACCATTAAC
RwhhapET28aXho1	AGCCGCTA CTCGAG TTAACGAATGAATTTCCATAC
FwtomBET28aNde1	TTTTCATATGATGGACGAATACTCAC
RwtomBEt28axho1	TTTTCTCGAGTTAACAAGACAAACTAAC
