# Supplementary material for: The Hha-TomB Toxin-Antitoxin System Shows Conditional Toxicity and Promotes Persister Cell Formation by Inhibiting Apoptosis-Like Death in S. Typhimurium
Source: Sci Rep. 2016 Dec 2;6:38204. doi: 10.1038/srep38204 (PMC5133643; doi:10.1038/srep38204)
Supplement: Supplementary Information [file srep38204-s1.pdf]

The Hha-TomB Toxin-Antitoxin System Shows Conditional Toxicity and Promotes Persister Cell Formation by Inhibiting Apoptosis-Like Death in *S.* Typhimurium

Sangeeta Jaiswal[1], Prajita Paul[1], Chandrashekhar Padhi[1], Shilpa Ray[1], Daniel Ryan[1], Shantoshini Dash[1], Mrutyunjay Suar[1#]

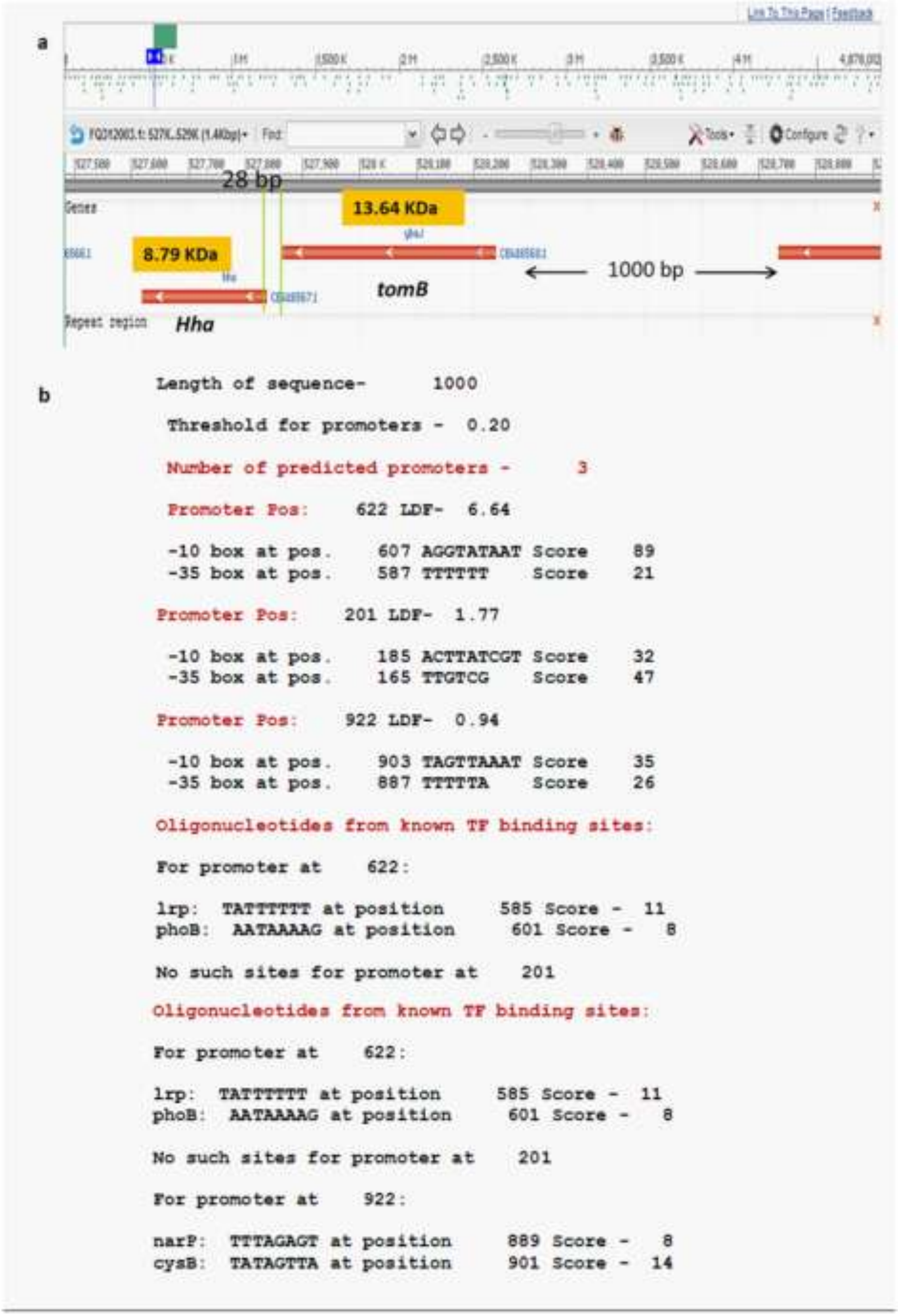

Fig S1: Genetic organization of Hha-TomB TA module and predicted promoters. (a). The genetic organization of Hha-TomB TA system was retrived from NCBI. The genes encoding Hha and TomB are present on the complementary strand. *tomB* is present upstream of *hha* and separation between the genes is 28 bp. **(b).** The BPROM program predicted three putative promoters with -35 and -10 sequences and transcription factor binding sites for each promoter.

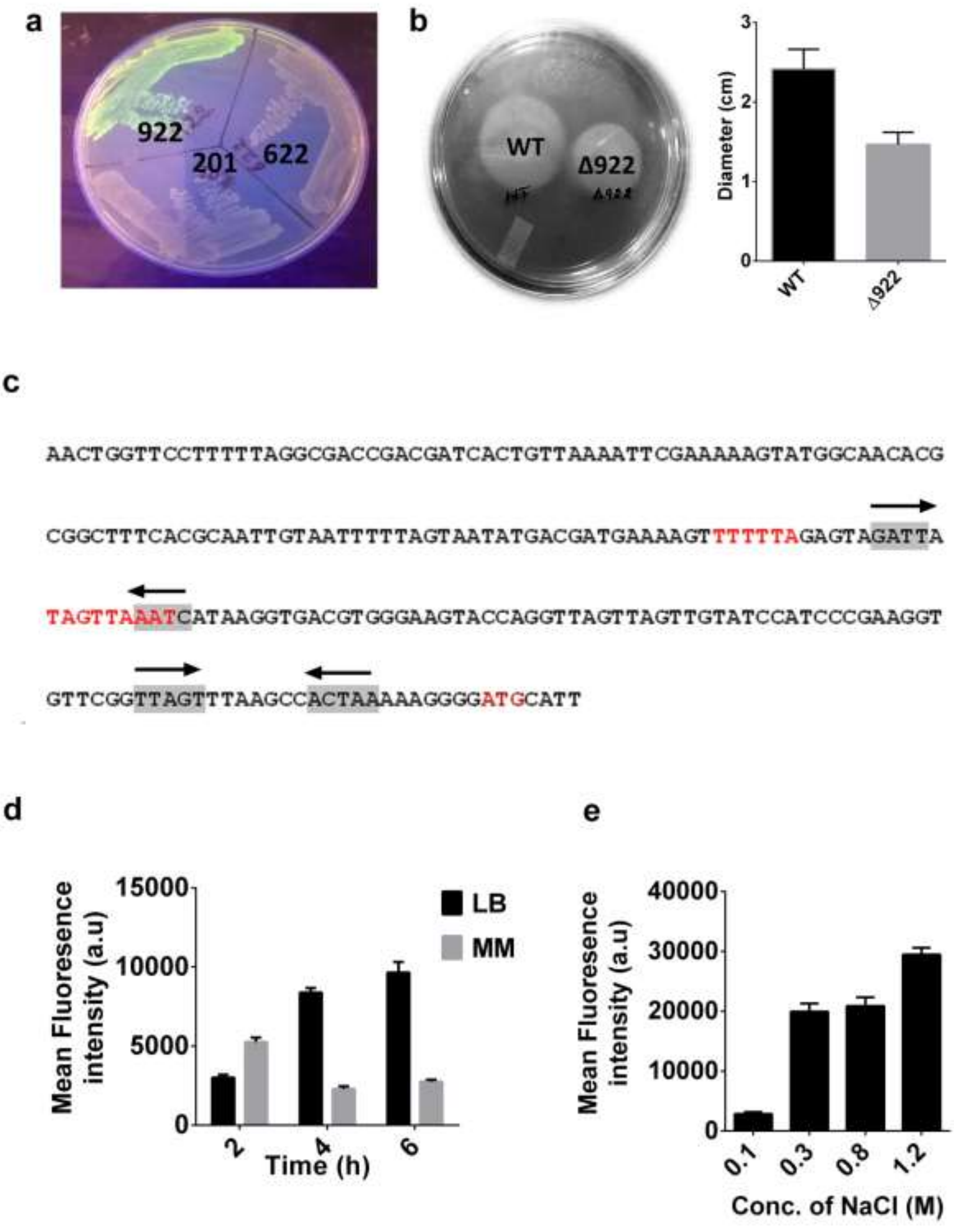

Fig S2: Identification and trancriptional regulation of p922 promoter. **(a)** Predicted promoters were cloned in a promoterless GFP plasmid (pM968) and visualised under UV. Only p922 promoter expressed GFP. **(b)** Motility assay of wild-type and p922 deletion

mutant and diameter of zone of motility. Wild-type *S.* Typhimurium, its isogenic mutants and complemented strain were grown overnight in LB supplemented with appropriate concentration of antibiotics and IPTG. 1 µl of overnight culture was pierce were placed at the center of the agar plate containing 0.3% agar and incubated for 8 h. The image is the representative of three independent experiments **(c)**. Sequence of cloned p922 promoter with putative transcriptional start site. -35 and -10 sequences are boxed. Palindromic sequences have been shown by opposite arrows. **(d)** p922 promoter construct were transformed into WT and the expression of GFP was analysed at indicated time points after growth in LB and minimal media. In LB, GFP expression increased with time while in minimal media it decreased. Furthermore, at 4 and 6 h GFP expression in minimal media was lower than LB. **(e)** Wild-type *S.* Typhimurium harbouring p922 GFP construct was grown in presence of indicated salt concentrations. With increasing salt concentration, GFP expression increased.

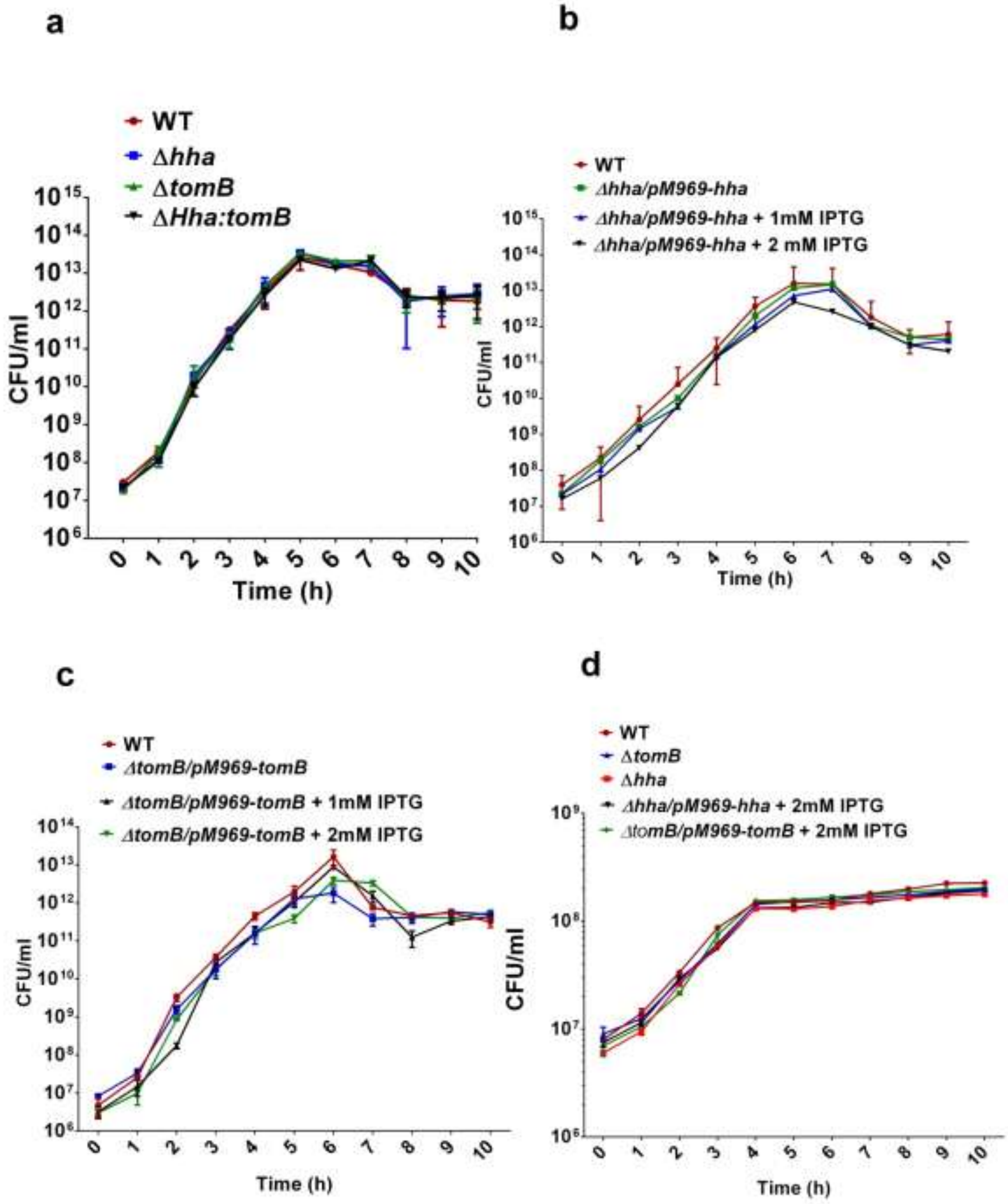

Fig S3: Analysis of growth of Wild type *S.* Typhimurium, Δhha, Δ*tomB* and their complemented strains. (a) WT, Δ*hha*, Δ*tomB*, Δ*hha:tomB* were grown in LB at 37°C, 150 rpm. At indicated times 100 µl of culture was withdrawn and appropriate dilutions were plated on LB agar plate supplemented with appropriate antibiotics. **(b)** *hha* gene was cloned under an inducible promoter in a plasmid and transformed into Δ*hha*. Growth of wild-type

and complemented strain was analyzed in LB after induction with 1 mM and 2 mM IPTG. **(c)** *tomB* gene was cloned under an inducible promoter in a plasmid and transformed into ΔtomB. Growth of wild-type complemented strain was analyzed in LB after induction with 1 mM and 2 mM IPTG. **(d).** WT, Δ*hha* and Δ*tomB* were grown in minimal media at 37°C, 150 rpm. Complemented strains were grown in presence of 2 mM IPTG in minimal media under above conditions. At indicated times 100 µl of culture was withdrawn and appropriate dilutions were plated on LB agar plate supplemented with appropriate. Data presented in the figure represent mean of three independent experiments.

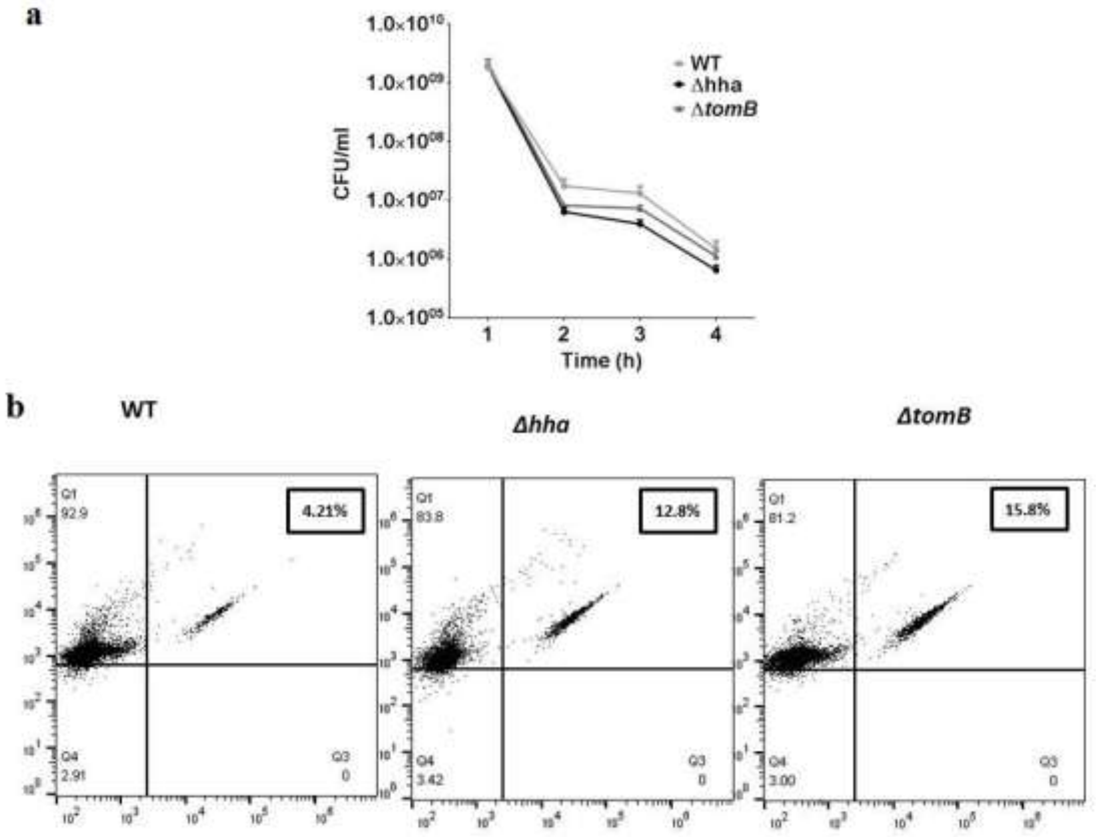

Fig S4: TUNEL assay: Overnight bacterial cells were subcultured for 2 h and treated with gentamicin (100 µg/ml). **(a)** 1 ml of culture was withdrawn at indicated time points and viable cells were determined by plating serial dilutions. **(b)** 1 ml of culture was withdrawn at

4 h post treatment and TUNEL assay was performed using In Situ Direct DNA Fragmentation Assay Kit and fluorescence intensity was measured by flow cytometry. The experiment was performed three times.

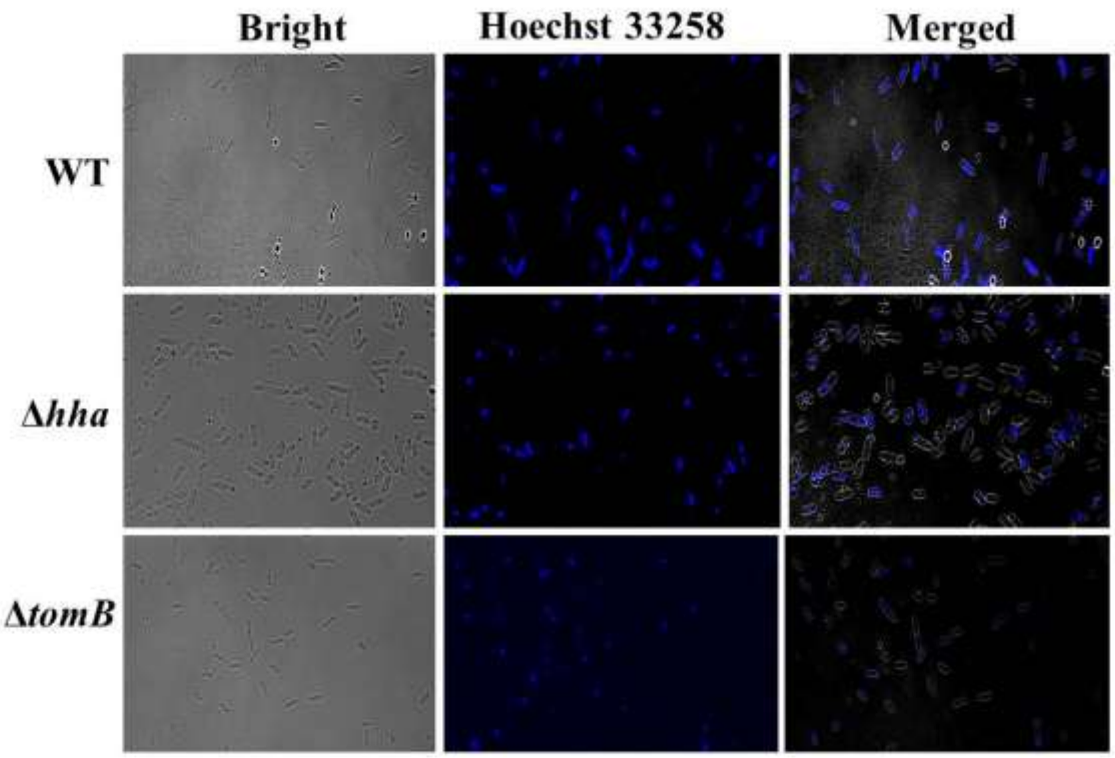

Fig S5: Structural changes in chromosome following gentamicin treatment. Overnight bacterial cells were subcultured for 2 h and treated with gentamicin (100 µg/ml). 1 ml of culture was withdrawn at 4 h post treatment, stained with Hoechst 33258 dye and visualised under fluorescence microscope (Olympus BX61, ImagePro ExpressTM,). Images were processed by ImageJ.

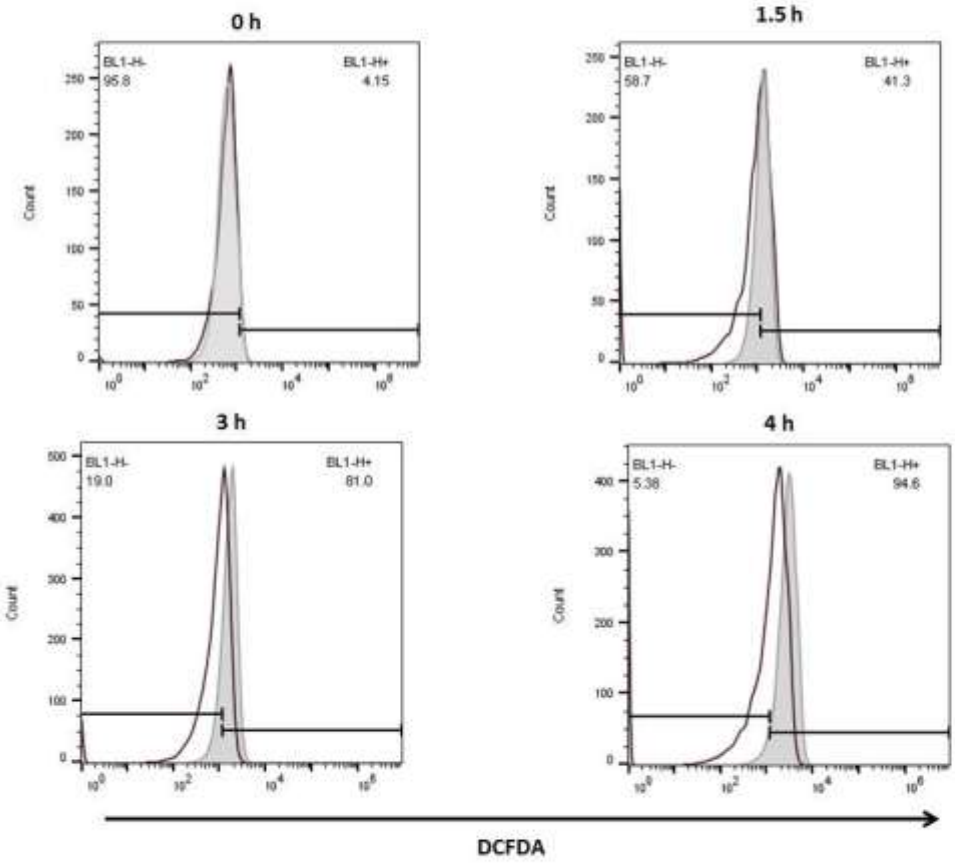

Fig S6: ROS production. Overnight grown bacterial cells were subcultured for 2 h and treated with gentamicin (100 µg/ml). 1 ml of culture was withdrawn at indicated time points and DCFDA dye was added at 10µM concentration. Fluorescence after 30 min was measured by flow cytometry.
